# Scandinavian entry points to social medicine and postcolonial health: Karl Evang and Halfdan Mahler in India

**DOI:** 10.1017/mdh.2023.7

**Published:** 2023-01

**Authors:** Sunniva Engh, Niels Brimnes

**Affiliations:** 1 Department of Archaeology, Conservation and History, University of Oslo, PO Box 1008 Blindern, N-0315 Oslo, Norway; 2 Department of History and Classical Studies, Aarhus University, Jens Chr. Skou’s Vej 5, DK-8000 Aarhus C, Denmark

**Keywords:** Social Medicine, Karl Evang, Halfdan Mahler, World Health Organisation, India, Postcolonial Health

## Abstract

Our contributions examine the Norwegian Karl Evang's (1901-1981) and the Dane Halfdan Mahler's (1923-2016) participation in international health co-operation facilitated by the World Health Organization (WHO) in India in the 1950s. While Evang’s was a hectic, but relatively short visit as part of a WHO visiting team of medical scientists in 1953, Mahler’s spanned the entire decade on assignments as WHO medical officer to tuberculosis control projects. Mahler’s name should be familiar to researchers of international health as the Director-General of the WHO 1973-88, and for his promotion of primary health care through the 1978 Alma-Ata Declaration. Evang, Norway’s Director of Health 1938-72, was also a key figure in international health in the mid-twentieth century as one of the original instigators of the WHO, and a participant in much of its early work.

A core theme is the place of social medicine, both in Evang’s and Mahler’s work, and within the WHO and its navigation of complex postcolonial settings in the 1950s. Investigating cross-regional encounters and circulations of social medicine ideas between Evang and Mahler and their Indian interlocutors as well as international WHO staff members, we ask what the role of social medicine was in international health in the early post-war period. Researchers have found that social medicine had its heyday during the 1930s and 1940s, and that a technology-focused, vertical approach became dominant soon after the war. In contrast, we suggest that continued circulation of social medical ideas points towards a more complicated picture.

Our contributions to this issue of *Medical History* investigate specific episodes in the careers of the Norwegian Karl Evang (1901–1981) and the Dane Halfdan Mahler (1923–2016). More specifically, we examine Evang’s and Mahler’s participation in international health cooperation facilitated by the World Health Organization (WHO) in the 1950s. Both episodes took place in India but differed in character and duration. While Evang’s was a hectic but relatively short visit as part of a WHO visiting team of medical scientists in 1953, Mahler’s spanned the entire decade on assignments as WHO medical officer to tuberculosis control projects. Halfdan Mahler’s name should be familiar to researchers of international health as the Director–General of the WHO in the years 1973–88, and for his promotion of primary health care, particularly through the 1978 Alma-Ata Declaration. Karl Evang, Norway’s Director of Health 1938–72, may be less well-known, but Evang was a crucial figure in international health in the mid-twentieth century; he was one of the original instigators of the WHO, and a participant in much of its early work.

Now, what makes the work of these two Scandinavian medical doctors, their participation in international health work, and their encounters with India’s medical scene relevant and fruitful cases for historical enquiry? Histories of Western medical men and their adventures in newly independent countries can easily smack of simplistic and celebratory tales of great white men and their institutions, seemingly obsolete. Indeed, Sunil Amrith has argued that for many years, the history of organized international health was written from a Western point of view, slanted towards the views of the great powers, and focused on the experiences of the League of Nations Health Organization and the World Health Organization. Thus, the resulting histories were often ‘a tale of progress, of heroic doctors and enlightened administrators.’^1^ With the history of medicine being influenced by post-structuralist scholarship, and with the increasing attention to asymmetries related to imperialism and power, gender, and social and economic inequalities, research on international health is undergoing something of a ‘reorientation of spatial horizons’, in Amrith’s words, where international health histories are progressively seen from outside the traditional power centres, and written, instead, from the perspectives of Asia, Latin America, and Africa.^2^ We argue that in such a reorientation of the narrative about international health cooperation, the history of Scandinavian actors’ involvement in international health also has a role to play. Their inclusion may help continue and widen international health histories’ reorientation along spatial horizons, investigating these histories from a slightly different standpoint: the Scandinavian one. Moreover, we find that these cases are necessary for the development of a truly global history, including the experiences of a greater range of historical actors, hailing from a variety of backgrounds. Thus, while Denmark and Norway were not among the major players in international health compared to, say, the United Kingdom or the United States of America, Evang and Mahler’s examples may still show us a broader involvement of regions and states in international health work.

What do we already know about Scandinavia in international health? Some research exists on Scandinavia in international health campaigns,^3^ Swedish health administrator Axel Höjer’s years in India,^4^ and Scandinavian aid to family planning efforts.^5^ In addition, public health co-operation between the Rockefeller Foundation and Scandinavian countries has been increasingly explored over the past few years.^6^ Research on Scandinavia and international organisations such as the League of Nations and the United Nations has largely focused on the small states’ commitment to these organisations due to their quest for peace, international stability, and security; it has paid less attention to these organisations’ medical work.^7^ Historical explorations have thus often retained a national or organisational focus, and Scandinavian countries’ roles in organised international health has been examined to a very limited extent.

Our articles build on primary sources from Danish, Norwegian, WHO and UNICEF archives, as well as literature from several historiographical fields, aiming to situate Evang and Mahler within international health, moving beyond strictly national or institutional frameworks, and rather investigating their transnational, cross-regional interactions. Highlighting intellectual exchanges between our protagonists and their counterparts both in India and in the WHO system, we hope our articles may pertain to discussions of India as a receptive but certainly also generative site of ideas and political practice in health, and to discussions of the WHO as a site for international governance.

In contrast to research that argues forms of Scandinavian exceptionalism in international politics,^8^ our focus on two Scandinavian doctors is thus not intended to investigate a particular Scandinavian way of conducting international health, but rather seeing how Evang and Mahler participated in international health politics and practice. At the same time, however, there is one aspect of their work which one could easily be tempted to relate to their ‘Scandinavianness’: social medicine.

A core theme in both our articles is the place of social medicine, both in Evang’s and Mahler’s work, but more importantly, within the WHO and its navigation of complex postcolonial settings in the 1950s. Investigating cross-regional encounters and circulations of social medicine ideas between Evang and Mahler and their Indian interlocutors, as well as international WHO staff members, we ask what the role of social medicine was in international health work in the early post-war period. Researchers have found that social medicine had its heyday during the 1930s and 1940s, but that social medical ideas were given little prominence in the WHO, as a technology-focused, vertical approach became dominant in the early post-war period, particularly following the Soviet Union’s 1949 exit, and the dominance of American interests ensued.^9^ In contrast, using Mahler’s and Evang’s experiences in international health work in India as our cases, we suggest that continued circulation of social medical ideas points towards a more complicated picture.

Finally, this focus on social medicine warrants a few facts about our protagonists. Evang and Mahler shared some basic traits: both were Scandinavian, and both were or became committed to social medicine. In addition, both had a particular interest in India as a site for medical work, and both had long-lasting connections to the World Health Organization. But here, the similarities end. While Evang was one of the pioneers of the WHO and was central in its early work, his main career was at home rather than in international health; he was Norway’s Director of Health 1938–1972. As such, Evang had immense influence on Norway’s health administration, particularly its rebuilding following World War II, and more precisely, its development into a modern welfare state. Mahler, on the other hand, spent the vast majority of his career within the WHO, including three full terms as Director–General and thus had a considerable, long-lasting influence on the organisation’s work.^10^

Another point of difference is Evang’s versus Mahler’s relation to social medicine. Whilst both were committed to social medicine as a direction in health, Evang first encountered social medicine whilst a student at the University of Oslo, immersed himself in the writings of Alfred Grotjahn, and connected with the German association for socialist physicians. Evang became a key promoter of social medicine in Scandinavia, establishing socialist physicians’ associations and publishing widely. Evang thus experienced and practiced social medicine in its heyday, also building on Grotjahn’s core ideas in his visions for Norway’s post-war health system. More to the point, when Evang travelled to India on behalf of the WHO in 1953, social medicine would have been one of Evang’s main influences from a professional medical point of view, one of the tools which he brought to India in his ‘medical toolkit’, so to speak. In Mahler’s formal medical education, in contrast, social medicine was not particularly emphasised. Thus, Mahler arguably encountered and was inspired by social medicine during his work in India, and he brought this experience back with him to the WHO Headquarters in Geneva.

In this way we offer two related but also quite different trajectories. Karl Evang’s story is about a strong-minded, self-confident health bureaucrat who came to India at the height of his career, with firm ideas about social medicine. Evang possibly assumed his Indian counterparts would be receptive to his ideas, but he found that their agendas at times clashed with his. Halfdan Mahler’s story is about a young, inexperienced doctor in a formative phase in his life and career, who discovered India as a site for inspiration and learning. These differences aside, however, we argue that Evang’s and Mahler’s encounters with the Indian medical scene, set within the WHO’s health governance efforts, were key experiences for both protagonists. We thus offer our analyses as contributions to a more complete and truly global history of health in the postcolonial period.

## Footnotes

1 Sunil Amrith ‘Internationalising Health in the Twentieth Century’, in G. Sluga and P. Clavin (eds), Internationalisms. A Twentieth-Century History (Cambridge: Cambridge University Press, 2017), 245 .

2 See e.g. Sunil Amrith , Decolonizing International Health. India and Southeast Asia, 1930–65 (London: Palgrave, 2006); A. Raza , F. Roy and B. Zachariah (eds), The Internationalist Moment: South Asia, Worlds, and World Views, 1917-39, (New Delhi: Sage, 2015); Sanjoy Bhattacharya , ‘The World Health Organization and global smallpox eradication’, Journal of Epidemiology and Community Health, 62, 10 (2008), 909–121879104910.1136/jech.2006.055590PMC2602749; Sanjoy Bhattacharya ‘International Health and the Limits of its Global Influence: Bhutan and the Worldwide Smallpox Eradication Programme’, Medical History, 57, 4 (2013), 461–862406991310.1017/mdh.2013.63PMC3865968; David Arnold , Colonizing the Body: State Medicine and Epidemic Disease in Nineteenth-Century India (Berkeley: University of California Press, 1993); Hellen Tilley , Africa as a Living Laboratory: Empire, Development, and the Problem of Scientific Knowledge, 1870–1950 (Chicago: University of Chicago Press, 2011).

3

Niels Brimnes , ‘Vikings against Tuberculosis: The International Tuberculosis Campaign in India, 1948-1951’, Bulletin of the History of Medicine, 81, 2 (2007), 407–430 1784472210.1353/bhm.2007.0022; Niels Brimnes , Languished Hopes . Tuberculosis, the State and International Assistance in Twentieth-century India, (New Delhi: Orient BlackSwan, 2016).

4 Annika Berg, *Den gränslösa hälsan. Signe och Axel Höjer, folkhälsan och expertisen* (PhD thesis: Uppsala University, 2009).

5 Sunniva Engh, *Population Control in the 20th Century: Scandinavian Aid to the Indian Family Planning Programme 1960–1995* (DPhil thesis: University of Oxford, 2006); Sunniva Engh , ‘From northern feminists to southern women. Scandinavian population aid to India’, in H. Ø. Pharo and M. P. Fraser (eds), The Aid Rush. Aid Regimes in Northern Europe during the Cold War, (Oslo: Unipub, 2008) 253–83; Annika Berg , ‘A Suitable Country: The Relationship between Sweden’s Interwar Population Policy and Family Planning in Postindependence India’, Berichte zur Wissenschaftsgeschichte, 33 (2010), 297–320 2146614410.1002/bewi.201001470.

6

Henriette Buus , Indretning og efterretning. Rockefeller Foundations inflydelse på den danske velfærdsstat 1920–1970 (Copenhagen: Museum Tusculanum Press, 2008); K. Petersen and H. Buus , ‘Americanization of the Danish welfare state? Traces of American influences on health and social policies in Denmark’, in K. Petersen , J. Stewart and M. Kuur Sørensen (eds), American Foundations and the European Welfare States (Odense: University Press of Southern Denmark, 2013), 107–34; Sunniva Engh , ‘Rockefeller Foundation og etableringen av Statens institutt for folkehelsen. Idéer, investeringer og institusjonsbygging i internasjonal helse, 1923–1935’, Michael, 16, 1 (2019), 10–35 ; Sunniva Engh , ‘Transatlantiske forbindelser i mellomkrigstidens helsearbeid: Rockefeller Foundation, public health og norsk sykepleierutdanning’, in B. Frydenlund , T. Hamre and A. Avelin (eds), Transatlantiske forbindelser. Norsk-amerikanske relasjoner gjennom 200 år (Oslo: Scandinavian Academic Press, 2021), 99–134; Sunniva Engh, ‘The Rockefeller Foundation, Scandinavian aid agencies and the “population explosion”’, in K. Petersen, J. Stewart and M. Kuur Sørensen, *op.cit.*, 181–202.

7

Karen Gram-Skjoldager , Haakon Andreas Ikonomou and Torsten Kahlert , ‘Scandinavians and the League of Nations Secretariat, 1919–1946’, Scandinavian Journal of History, 44, 4 (2019), 454–83.

8 See e.g. Christine Ingebritsen , ‘Norm Entrepreneurs. Scandinavia’s Role in World Politics’, Cooperation and Conflict, 37, 1 (2002), 11–23 ; Hans Mouritzen , ‘The Nordic Model as a Foreign Policy Instrument: Its Rise and Fall’, Journal of Peace Research, 32, 1 (1995), 9–21 ; David Lumsdaine , Moral Vision in International Politics: The Foreign Aid Regime 1949–1989 (Princeton, NJ: Princeton University Press, 1993).

9

Iris Borowy , Coming to Terms with World Health. The League of Nations Health Organisation 1921–1946, (Frankfurt am Main: Peter Lang, 2009), 21–2; Randall Packard , A History of Global Health. Interventions into the Lives of Other Peoples (Baltimore: Johns Hopkins University Press, 2016), 91–131 ; Sunil Amrith, op. cit. (note 2), 47–56, 93, 189–90; Marcos Cueto , Theodore M. Brown and Elizabeth Fee , The World Health Organization. A History (Cambridge: Cambridge University Press, 2019), 62–4; Niels Brimnes , ‘Rallying around the magic wand: visions of social medicine, public health and disease control in India 1946–57’, in I. Borowy and B. Harris (eds) Health and Development. Yearbook for the History of Global Development, Vol. 2. (Oldenbourg: De Gruyter, 2023), 175–99.

10 After stepping down as Director–General, Mahler spent seven years as Director of IPPF (1988–1995). His tenure with WHO lasted 47 years (1951–1988, except for at brief break in the mid 1950s).

